# Polyhydroxybutyrate (PHB): Production, Properties, Modification Strategies, Additive Manufacturing, Biodegradation, and Applications

**DOI:** 10.3390/ma19143115

**Published:** 2026-07-20

**Authors:** Bairavi Sanjeevi, Duncan E. Cree

**Affiliations:** Department of Mechanical Engineering, McMaster University, Hamilton, ON L8S 4L7, Canada; sanjeevb@mcmaster.ca

**Keywords:** polyhydroxybutyrate (PHB), polyhydroxyalkanoates (PHAs), biodegradable polymers, bioplastics, additive manufacturing, fused deposition modeling (FDM), polymer composites, biodegradation

## Abstract

Growing environmental concerns over petroleum-based plastics have increased interest in sustainable and biodegradable alternatives such as polyhydroxybutyrate (PHB). PHB is a naturally produced biopolymer synthesized by microorganisms and is widely recognized for its biodegradability, biocompatibility, renewability, and thermoplastic properties. Despite these advantages, PHB use remains limited by brittleness, high crystallinity, low thermal stability, a narrow processing window, and high production costs. This review discusses the production, properties, biodegradation behavior, and applications of PHB, with a focus on strategies to improve its performance. Modification approaches, including copolymerization, polymer blending, filler reinforcement, plasticization, and hybrid composite formulation, are critically reviewed to evaluate their effects on the thermal, mechanical, and processing behavior of PHB-based materials. The review also highlights recent developments in additive manufacturing, particularly fused deposition modeling/fused filament fabrication (FDM/FFF) for the extrusion of biodegradable PHB composite filaments. In addition, the biodegradation of PHB under various environmental conditions, including soil, compost, freshwater, marine, aerobic, and anaerobic environments, are discussed. Current challenges, research gaps, commercialization barriers, and future opportunities related to sustainable feedstocks, advanced composites, additive manufacturing, and circular economy integration are addressed. Overall, PHB shows strong potential as a sustainable alternative for packaging, biomedical, agricultural, and three-dimensional (3D) printing applications.

## 1. Introduction

Conventional polymers, which are predominantly manufactured from fossil-derived feedstocks, pose significant environmental concerns because they generate persistent plastic waste, contribute to greenhouse gas emissions, and negatively affect ecosystems [1]. The growing use of petroleum-based plastics has also resulted in widespread microplastic contamination in soil, air, and aquatic environments [2,3]. Consequently, increasing research efforts have focused on developing bio-based polymers as sustainable alternatives because of their renewable origin, lower environmental impact, and, in many cases, their biodegradability [4].

Among these materials, polyhydroxyalkanoates (PHAs) have attracted considerable attention due to their biodegradability, biocompatibility, and production from renewable resources. Poly(3-hydroxybutyrate) (PHB), the simplest and most extensively studied member of the PHA family, has emerged as a promising substitute for conventional plastics because of its thermoplastic properties and potential to support circular economy initiatives [5,6,7,8]. However, its broader industrial application remains limited by high crystallinity, brittleness, low thermal stability, a narrow processing window, and relatively high production costs [4,9,10,11,12]. To overcome these limitations, numerous studies have investigated copolymerization, polymer blending, plasticization, and the incorporation of fillers and nanofillers to improve the mechanical, thermal, and processing performance of PHB while maintaining its biodegradability [13,14,15,16].

Recent advances have further expanded the application of PHB into sustainable packaging, biomedical engineering, agriculture, and additive manufacturing, particularly fused deposition modeling (FDM), where material formulation and processing conditions strongly influence printability and final component performance [13,17,18,19]. Previous reviews have examined PHB production and sustainability [20], biodegradable polymers in healthcare and additive manufacturing [21], PHA functionalization [22], microalgae-based PHA production [23], agricultural applications [24], and biomedical uses [25]. However, comparatively limited attention has been given to systematically relating PHB blend composition, composite filament development, FDM processing parameters, and their influence on the thermal and mechanical performance of printed components.

This review was developed through an extensive evaluation of recent studies on the production, physicochemical properties, modification strategies, biodegradation, and industrial applications of PHB. Special focus was placed on research studies involving PHB-based blends, fillers, and plasticizers for additive manufacturing. In addition, this review compares the formulation of PHB blends and composites, filament fabrication conditions, fused deposition modeling (FDM) processing parameters, and their effects on printability, thermal behavior, and mechanical performance. By integrating these aspects with PHB biosynthesis, biodegradation, and application studies, this article provides a comprehensive overview of current advances, identifies existing challenges, and highlights future research directions for the development and commercialization of PHB-based materials.

## 2. Background on Polymer Classification and Environmental Impact

### 2.1. Classification of Polymers

Polymers can be categorized using various criteria, including their source (natural or synthetic), behavior under heat and mechanical stress, intended application, methods of synthesis, as well as their chain architecture, molecular arrangement, and structural configuration [26]. Natural biopolymers are the most common organic materials in the earth’s biosphere. They are naturally formed polymers synthesized by the cells of living organisms, including plants and animals [27]. In contrast, synthetic polymers are man-made materials created through chemical processes to serve specific purposes or fulfill particular functional requirements [28]. Although they have traditionally been manufactured from petroleum-based feedstocks, advances in sustainable polymer technologies have enabled the use of wholly or partially bio-based and renewable feedstocks for their production [29,30].

**Figure 1:**
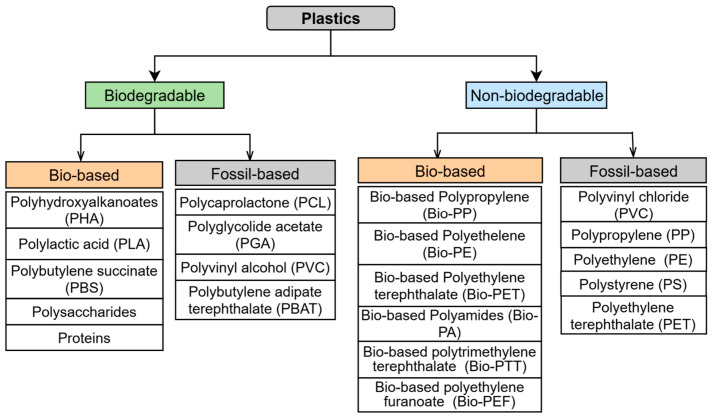
Classification of biodegradable and non-biodegradable plastics based on feedstock origin and degradability. Developed by the authors based on information synthesized from [30,31].

Bioplastics include different types of polymer materials and are usually classified based on their source and biodegradability. They can be grouped into three types: (i) bio-based and biodegradable plastics made from renewable resources like plants and are able to decompose naturally, (ii) biobased but non-biodegradable plastics derived from biomass are not easily broken down by biological processes such as fungi or bacteria, and (iii) fossil-based biodegradable plastics made from petroleum sources but still capable of biodegrading under suitable conditions [31]. Figure 1 illustrates the classification of plastics according to their biodegradability and feedstock origin [30,31]. Altogether, both synthetic and biological polymers are indispensable materials. Their structural diversity and functional adaptability not only support various biological processes but also enable innovative applications in science, engineering, and technology.

### 2.2. Plastics vs. Polymers

From a functional standpoint, plastics refer to a group of synthetic or semisynthetic materials derived from polymers, designed to be shaped or molded during production. They are distinguished by their ability to maintain form and size under applied stress, making them suitable for a wide range of general-purpose uses. Within the broader class of polymers, plastics represent a specific subset defined primarily by their end-use applications. Because plastics are largely made from polymers, the terms are frequently confused and used interchangeably in everyday language [26]. Plastics are composed of polymers, but not every polymer is classified as a plastic [32]. Polymers can be produced from a variety of feedstocks. Bio-based polymers are derived wholly or partly from biological resources, such as lignocellulosic biomass, algae, microorganisms, cellulose, and starch [33]. In contrast, conventional plastics are generally produced from fossil-based carbon, whereas bio-based plastics may be manufactured partially or entirely from renewable biomass [34]. Plastics therefore represent a specific category of polymer-based materials, while polymers form a broader group that includes both plastic and non-plastic materials.

Thermoplastics are widely used due to their adaptability, moderate strength, maintaining stability under different temperatures (though they soften or melt when heat exceeds their glass transition temperature (*T*_g_) or melting temperature (*T*_m_), respectively), resistance to chemicals, corrosion, and are light weight [28,35]. Additives are often incorporated into plastics to make them lighter, more flexible, and mechanically stronger, thereby improving their performance for specific applications [36,37]. Synthetic plastics such as polyethylene (LDPE and HDPE), polypropylene (PP), polystyrene (PS), polyvinyl chloride (PVC), polyethylene terephthalate (PET), and polyurethane (PUR) are among the most widely produced polymers. Together, the first five types represent over 90% of global plastic production by weight [38,39]. Packaging uses most plastics, followed by construction, automotive, electronics, textiles, agriculture, and other sectors. Long-lasting products, especially in construction, form the largest plastic stocks, while packaging and agriculture contribute less due to shorter lifetimes [40,41].

### 2.3. Environmental Concerns of Plastics

Despite their functional advantages, synthetic plastics negatively affect both human health and the environment at every stage of their life cycle [42,43,44]. Plastic pollution has become a serious worldwide concern, with an estimated 19–23 million tonnes of plastic waste entering aquatic environments each year and contaminating rivers, lakes, and oceans [45]. They contribute to pollution across marine, soil, and atmospheric systems due to their slow and variable degradation rates [36,46]. Depending on the plastic type, they may persist in the environment for years or even decades [47]. Since conventional “synthetic” plastics do not easily degrade under natural conditions, they accumulate in the environment over time, leading to widespread and persistent pollution [48,49]. This can lead to soil infertility, making the land unsuitable for crop growth [50].

The degradation of plastics occurs through multiple pathways involving physical, chemical, and biological agents, resulting in fragmentation into macro-plastics (larger than 5 mm), large microplastics (between 0.3 mm and 5 mm), and small microplastics (smaller than 0.3 mm, including nano-plastics) [49,51]. Microplastics can disrupt aquatic ecosystems by reducing light penetration, lowering oxygen levels, and adhering to organisms, eventually accumulating within their tissues. In addition, their transfer to humans through seafood consumption poses serious health concerns [36]. Given their presence throughout the food chains, further research is urgently needed to understand their distribution, impacts, and behavior, particularly in freshwater systems [36,52].

Recent human tissue studies further suggest that microplastics and nano-plastics may not only affect aquatic organisms but may also reach and accumulate in human brain tissue. In one study, microplastics were detected in the olfactory bulb of deceased individuals, where synthetic polymer particles and fibers were found in 8 out of 15 cases. Polypropylene was the most common polymer identified, accounting for 43.8% of the detected plastics, followed by nylon and other polymer types. This finding suggests that the olfactory pathway may act as a possible route for airborne plastic particles to enter the brain [53]. Another study reported that micro- and nano-plastic concentrations in brain tissue were 7–30 times higher than those found in liver or kidney samples, with even greater accumulation observed in dementia cases. Most of these particles were identified as polyethylene and appeared as nano-plastic shards or flakes. Although these findings do not confirm a direct relationship between plastic particles and neurodegenerative disease, they raise concern about the potential neurotoxic effects of microplastics and nano-plastics and highlight the need for further investigation into their entry pathways, accumulation, and long-term effects on human brain health [54].

### 2.4. Global Plastic Production and Waste Generation

**Figure 2:**
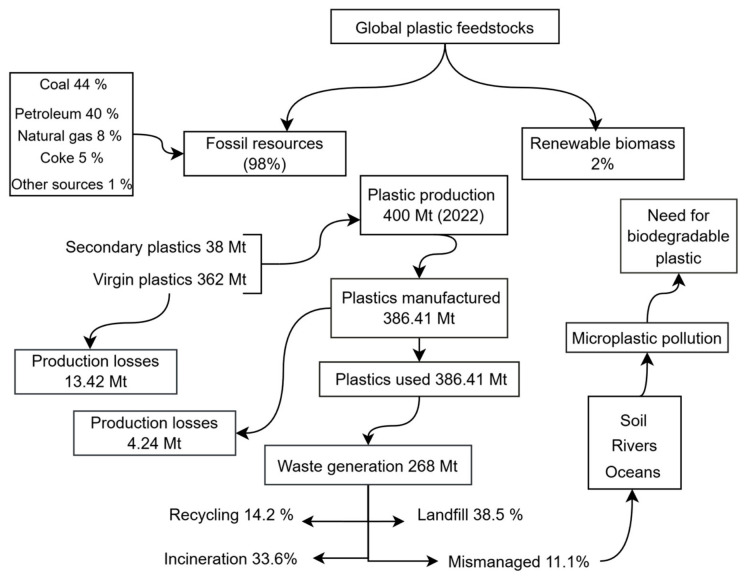
Schematic representation of global plastic production and waste management in 2022 based on the reported mass-flow data. Redrawn by the authors using data from [40]. Note: minor discrepancies in values are due to rounding during mass-balance calculations.

Although plastics are essential in many aspects of modern life, the widespread use of non-degradable plastics has led to major environmental challenges. In 2016, around 219 million tonnes of municipal solid waste plastic reached its end-of-life stage worldwide. Of this amount, only 31 Mt/year was recycled, whereas 97 Mt/year was disposed of and 91 Mt/year was not properly managed. Mismanaged plastic waste commonly ends up in open burning, dumpsites, and aquatic or terrestrial environments [55]. For example, in 2022, global plastic output was approximately 400 Mt, consisting of about 362 Mt of newly produced virgin plastics and roughly 38 Mt obtained from mechanical recycling. Nearly 98% of virgin plastics originated from fossil-based resources, including coal (44%), petroleum (40%), natural gas (8%), coke (5%), and other sources (1%), while only 2% were derived from renewable biomass. Production losses (e.g., rejected/defective parts, purgings, and runners) of virgin plastics were estimated at 13.42 Mt, leaving 348.52 Mt available for further processing. After including recycled inputs, total plastic manufacturing reached around 386.41 Mt, with an additional 4.24 Mt lost during processing. Consequently, approximately 382.12 Mt of plastics were put into use. Packaging represented the largest application sector, accounting for 158.04 Mt, followed by building and construction at 72.05 Mt. The remaining plastics were used in automotive applications (32.02 Mt), electrical and electronic products (28.02 Mt), household and textile products (28.01 Mt), agriculture (16.01 Mt), and other sectors (48.03 Mt). Global plastic waste generation in 2022 was estimated at 267.68 Mt. Figure 2 shows the distribution of global plastic waste management in 2022. Landfill disposal was the largest reported management pathway, accounting for 103.10 Mt, followed by incineration at 89.99 Mt. Smaller quantities were mechanically recycled (37.96 Mt) or classified as mismanaged waste (29.60 Mt). These flows correspond to approximately 38.5%, 33.6%, 14.2%, and 11.1% of total waste generation, respectively, and together represent about 97.4% of the reported total. The remaining difference reflects rounding and flow allocation within the mass-balance analysis reported in the original study [40]. Without effective end-of-life management, the accumulation of plastic waste will continue unchecked, leading to long-term environmental consequences across global ecosystems [42,56]. Between 2016 and 2022, global plastic waste generation increased by approximately 22% [40,55]. This growth shows that plastic waste continues to rise despite recycling and disposal efforts, highlighting the need for improved end-of-life management and sustainable alternatives.

### 2.5. Limitations of Plastic Waste Management and Need for Sustainable Alternatives

Plastic waste can be handled through several approaches, such as recycling, thermal treatment, and disposal in landfills, each with limitations [42]. Mechanical recycling requires clean and well-sorted input materials, as contamination and repeated processing can contribute to quality degradation over multiple recycling cycles [57]. Chemical methods produce high-quality results but are costly and energy-intensive. Physical recycling requires compatible solvents and complex equipment. Biological recycling is limited to less common biodegradable plastics, and energy recovery generates harmful waste without yielding reusable materials [58]. Plastic waste management is difficult because of contamination, poor sorting, material degradation, and limited recycling facilities. Therefore, sustainable and cost-effective methods are needed to reduce, reuse, and manage plastic waste [59].

Growing awareness about the environmental harm caused by conventional plastics has pushed many nations to explore greener alternatives like bioplastics [60]. Bioplastics play a key role in cutting down our reliance on fossil fuel–based materials. By shifting away from petrochemical resources, they help move production and consumption systems toward a more sustainable, circular model where materials are reused and waste is minimized [61]. Since nature cannot break down synthetic pollutants effectively, shifting to biodegradable options is becoming essential. With over 100 million tonnes of plastic produced each year, it is crucial to improve the methods we use to produce, recycle, and manage materials to prevent long-term environmental damage [62]. Therefore, the limitations of current plastic waste management methods highlight the need for materials that can be managed more effectively at the end of their life. In this context, understanding biodegradation is important because biodegradable polymers are often considered one possible route for reducing the long-term accumulation of plastic waste.

### 2.6. Biodegradation and End-of-Life Behavior of Biodegradable Polymers

Biodegradation is the breakdown of polymeric materials by microorganisms into simpler end products under suitable environmental conditions [63]. Biodegradable polymers can be treated through several end-of-life routes, including composting, recycling, enzymatic treatment, and anaerobic digestion [64]. However, biodegradation should not be confused with composting. While biodegradation occurs through microbial activity, composting provides controlled conditions that help speed up this process. As a result, compostable plastics may degrade efficiently in composting facilities where the environment is controlled, but may not break down easily in natural environments such as soil, water, or landfill. This distinction is important because misunderstanding compostable labels can lead to improper disposal and contribute to plastic pollution [65]. For instance, PHB degradation has been shown to improve under aerated conditions in the presence of certain fungal and bacterial strains, whereas PLA generally degrades more slowly, with stronger degradation mainly associated with *Penicillium chrysogenum* and *Bacillus subtilis* [66]. Environmental conditions strongly influence biodegradation. For example, temperature, moisture, oxygen availability, and microbial activity can accelerate or slow the process. PHB degradation in soil is particularly affected by temperature and moisture, whereas degradation in aquatic environments is often slower because oxygen availability and microbial activity are lower [67]. A recent comparison of PHB, PLA, and PBAT also found slower degradation in natural freshwater than in sludge-inoculated media [68]. Therefore, biodegradation depends on both polymer type and disposal conditions.

## 3. Biodegradable Polymers and Polyhydroxyalkanoates (PHAs)

### 3.1. Overview of PHAs

In recent years, researchers have focused on more sustainable alternatives, including engineering or high-performance biopolymers such as polyhydroxyalkanoates (PHAs), polylactic acid (PLA), starch-based blends [69], cellulose-derived plastic [70], polybutylene succinate (PBS), poly-ϵ-caprolactone (PCL), poly(butylene adipate-co-terephthalate) (PBAT) [71], and emerging biobased polyamides such as polyamide 11 (nylon-11) [72]. These materials can provide properties comparable to conventional petroleum-based plastics while offering improved sustainability. PHAs have gained significant interest due to their biodegradability and environmental friendliness [6,62]. Derived from renewable sources, PHAs are non-toxic, biocompatible, and possess several desirable physical properties such as high crystallinity, isotactic structure (stereoregularity), piezoelectricity, and water resistance. These qualities make them suitable for a wide range of applications, from packaging films and coated paper to disposable items like razors, diapers, and cosmetic containers [62], cups, straws, food packaging, and various single-use containers [7]. Moreover, PHAs can be synthesized from diverse feedstocks, including sugars [8], starches, plant oils, food waste [73], and industrial byproducts [8], enhancing their appeal as a sustainable material.

### 3.2. Types and Classification of PHAs

PHAs are classified into three types based on the number of carbon atoms in their monomer units. PHAs with up to five carbons are known as short-chain-length PHAs (scl-PHAs), those with six to fourteen carbon atoms as medium-chain-length PHAs (mcl-PHAs), and those with more than fourteen carbon atoms as long-chain-length PHAs (lcl-PHAs) [7]. The most common scl-PHA is poly(3-hydroxybutyrate) (PHB), also interchangeably used as P3HB, which is derived from the monomer R-3-hydroxybutyric acid and contains methyl side groups. PHB has been the most extensively studied member of the PHA family [6,7,8]. Another important scl-PHA is poly(4-hydroxybutyrate) (P4HB). Both PHB and P4HB are biosynthesized by various microorganisms, and their properties are influenced by differences in molecular structure, molecular weight, and carbon-chain arrangement [10].

**Figure 3:**
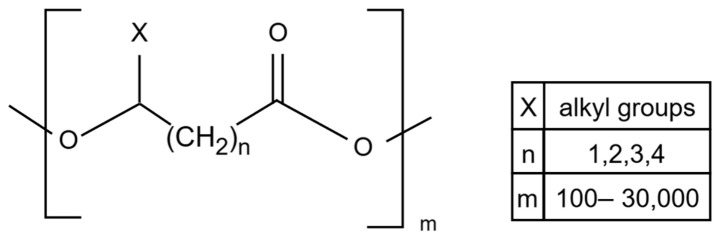
Structural formula of PHA redrawn by the authors [76].

Additionally, PHAs can contain hydroxyalkanoate monomer units such as 3-hydroxypropionate (3HP), 3-hydroxybutyrate (3HB), 3-hydroxyvalerate (3HV), 3-hydroxyhexanoate (3HHx), and 4-hydroxybutyrate (4HB). Examples of the corresponding polymers include poly(3-hydroxypropionate) [P(3HP)], poly(3-hydroxybutyrate) [P(3HB)], poly(4-hydroxybutyrate) [P(4HB)], and the copolymer poly(3-hydroxybutyrate-co-3-hydroxyvalerate) [P(3HB-co-3HV)] [74]. The numerical prefix in each monomer name indicates the position of the hydroxyl group along the carbon chain. Figure 3 presents the general repeating unit structure of PHAs, in which X represents the side group, n indicates the number of methylene (–CH_2_–) groups, and m represents the number of repeating units in the polymer chain, or the degree of polymerization [75,76].

## 4. PHB Production and Microbial Biosynthesis

### 4.1. Biosynthesis in Microorganisms

**Figure 4:**
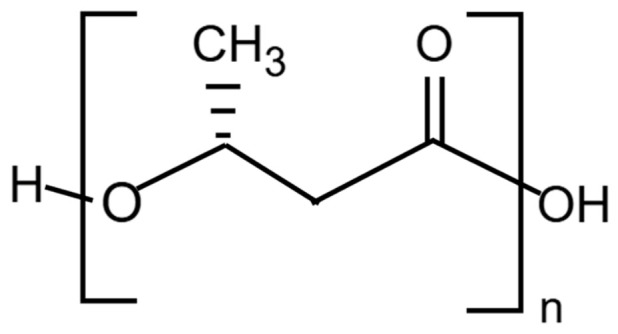
Structural formula of PHB redrawn by the authors [77].

PHB is a microbial polyester composed of repeating β-hydroxybutyrate units with the structure [77] as shown in Figure 4. Its role as a carbon and energy storage material is that it is synthesized by bacteria such as *Ralstonia*, *Vibrio*, *Halomonas*, *Pseudomonas*, and *Bacillus* under limited nitrogen, phosphorus, magnesium, or oxygen conditions with excess carbon availability [6,78]. In many microorganisms, PHB accumulates as intracellular granules within the cytoplasm, the fluid-filled region of the cell where metabolic activities occur and may account for up to 90% of the cell’s dry mass. This biological origin gives PHB two important advantages: it is produced from renewable resources and is biodegradable, positioning it as a sustainable alternative to petroleum-derived plastics. Large-scale production has been realized, and one of the most notable commercial products is Biopol^®^, a PHB/Polyhydroxyvalerate (PHV) copolymer [78,79].

PHB stands out from other biodegradable polymers as it has an exceptionally high crystallinity, which can reach 80%. In comparison with PLA, polyglycolic acid (PGA), PCL, and other co-polymers generally have a crystallinity range between 37 and 55%, while polyhydroxyoctanoate (PHO) remains much lower at 25% [79]. This high crystallinity contributes to PHB’s well-defined melting behavior and enhanced resistance to thermal decomposition, which is characterized by higher degradation onset temperatures, slower degradation kinetics, and improved flax fiber–matrix adhesion [79,80]. These features translate into superior stiffness and mechanical strength, making PHB particularly effective for composite reinforcement [79]. Beyond materials science, PHB has been found to play important physiological roles. For example, Rosetta Reusch [71] revealed that the different chain lengths in PHB polymers have distinct functions: medium chains contribute to ion channel formation in membranes, while shorter chains act as post-translational modifications of proteins [81]. Oligomeric PHB has also been linked to antioxidant activity and microbial growth promotion [82,83].

PHB has been detected across all kingdoms of life or parts thereof. It occurs naturally in the form of microorganisms such as *Escherichia coli* and baker’s yeast, in animals including human serum albumin, bovine aortic tissue, bovine serum albumin, and various organs (e.g., brain, lung, heart, liver) of porcine, sheep intestine, cat muscles, and snail visceral hump, as well as in plants such as spinach leaves, gorse, peanuts, beet stems, and spinach. Notably, PHB concentrations vary with health status in animals, suggesting a broader biological significance [78,82,83].

Bacteria that produce PHB can grow on many different feedstocks, ranging from sugars like glucose and sucrose to plant starch, vegetable oils, and even low-cost waste such as glycerol, dairy byproducts, food industry residues, or fruit peels. The type of feedstock used has a significant influence on both the cost of production and the sustainability of the process [84]. Overall, PHB is not only a promising biodegradable alternative to conventional plastics but also a biopolymer of structural, physiological, and evolutionary importance [78].

PHB has material properties comparable to synthetic polyethylene and polypropylene, but its wider use is restricted by high production costs influenced by microbial yield, substrates, fermentation, and purification. The production process involves three main steps: culture preparation, fermentation, and purification, with fermentation being the major contributor to the overall cost [85].

### 4.2. Biosynthetic Pathway

The first example of PHA was poly(3-hydroxybutyrate) P(3HB), which was identified in *Bacillus megaterium* by a French microbiologist, Maurice Lemoigne, in 1926 [86]. PHB production methods include bacterial fermentation, chemical synthesis via β-lactone polymerization, and the use of genetically modified plant cells [6].

**Figure 5:**
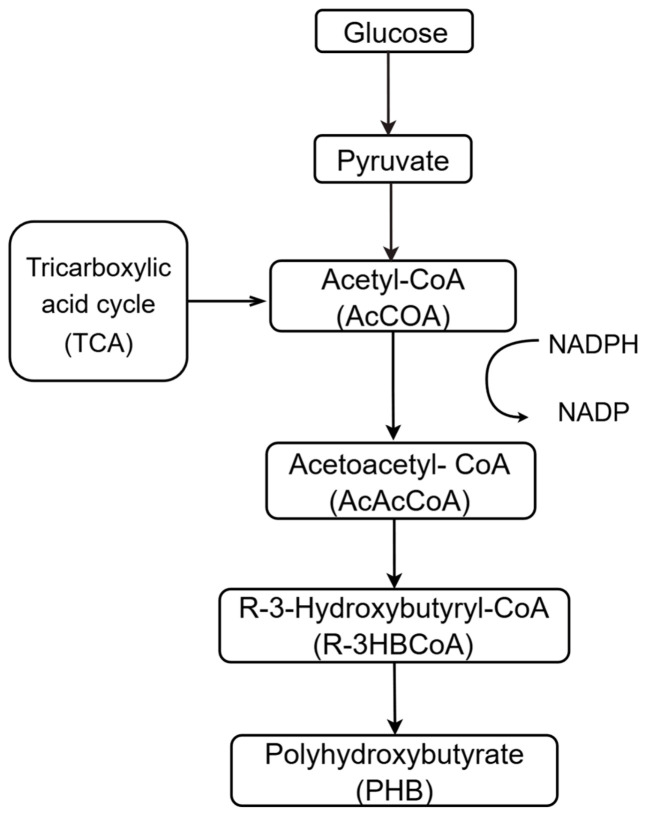
Flowchart for the engineered PHB biosynthetic pathway in *C. glutamicum* image redrawn by the authors [87].

PHB production methods include bacterial fermentation, chemical synthesis via β-lactone polymerization, and the use of genetically modified plant cells [6,78]. Figure 5 illustrates the heterologous PHB biosynthetic pathway derived from *Ralstonia eutropha* and engineered in *Corynebacterium glutamicum* (*C. glutamicum*) [87]. In this pathway, glucose is metabolized through a central carbon metabolism to produce pyruvate, which is subsequently converted into acetyl coenzyme A (acetyl-CoA) (AcCOA), the main precursor for PHB synthesis. The pathway involves three key enzymes encoded by the *phaA* (*β*-*ketothiolase*), *phaB* (*acetoacetyl*-*CoA* (AcAcCoA) *reductase*), and *phaC* (PHB *synthase*) genes. *PhaA* catalyzes the condensation of two acetyl-CoA molecules into acetoacetyl-CoA, which is subsequently reduced to R-3-hydroxybutyryl-CoA (R-3HBCoA) by the nicotinamide adenine dinucleotide phosphate (NADPH)-dependent enzyme *phaB*. During this reduction step, NADPH acts as a reducing cofactor and is oxidized to nicotinamide adenine dinucleotide phosphate (NADP^+^). Finally, *phaC* polymerizes R-3-hydroxybutyryl-CoA to form PHB. Optimization of central carbon metabolism in *C. glutamicum* was reported to improve PHB production from glucose and fructose under minimal medium conditions [87].

### 4.3. Reactor Design and Operating Conditions

PHB production can be carried out through the fermentation (fed-batch fermentation process) of sucrose, using *Azohydromonas australica*, in a stirred-tank reactor under nitrogen-limited conditions. The reactor operation is based on the following assumptions: carbon and nitrogen are the limiting substrates with an isothermal operation at 33 °C, with a constant pH of 7.0, and oxygen saturation with perfect mixing. In batch fermentation, all nutrients are supplied at the beginning of the process, and no additional substrate is added during cultivation. In contrast, fed-batch fermentation involves the controlled addition of nutrients throughout the fermentation period, allowing better regulation of microbial growth and substrate availability. The fed-batch (semi-open system) operation was found to improve yield and productivity, lower costs, and reduce CO_2_ emissions, energy, and water use compared with a batch mode. Depending on the microorganism, substrate, and operating conditions, PHB fermentation generally requires approximately 1–4 days, with an optimized fed-batch process being able to achieve maximum PHB production within about 36 h (closed system) [88].

For example, in a study, PHB was fermented for 42 h in a 3.7 L lab fermenter (1.5 L working volume) using 8% *v*/*v* preinoculum. Glucose (20 g/L) and glycerol (10, 20, 50 g/L) were tested as carbon sources under different conditions of temperature (30–33 °C), pH (5, 7, 9), and oxygen saturation (30–80%), where the stirring was maintained at 200–300 rpm with a steady airflow of 12 L/min. On a larger scale, glycerol proved to be more cost-effective and reduced production costs by 10–20% compared with using glucose, while crude glycerol, a byproduct of biodiesel production, showed a high valorization potential of 166.2% when used for PHB production [85].

## 5. PHB Properties and Performance

### 5.1. Molecular and Crystalline Structure

PHB is produced and stored inside bacterial cells as an intracellular polymer. In its native state within the cell, PHB is generally present in an amorphous form. However, after extraction using organic solvents, the polymer structure may become more ordered, resulting in a highly crystalline form [89]. PHB granules produced by *Bacillus megaterium* have been reported to show a spherical morphology under scanning electron microscopy (SEM), with particle sizes ranging from about 0.7 to 0.95 µm. Further examination using transmission electron microscopy (TEM) indicated that the granules were semi-crystalline and generally uniform in shape, with diameters of approximately 200–500 nm [5]. A related study showed PHB synthesized by *Bacillus megaterium* exhibited a relatively high molecular weight and low polydispersity, indicating a good polymer uniformity compared with synthetic polymers such as PE and PP [5]. Similarly, engineered *Cupriavidus necator H16* (Lgg-16) cultivated on molasses can produce PHB with molecular characteristics comparable to standard PHB, supporting the use of agricultural byproducts in a circular bioprocess [90]. In another study, *Azotobacter vinelandii N*-*15* grown in Burke’s medium supplemented with molasses produced PHB with a high purity of 98.9% and a crystallinity of 73%. PHB produced by *Azotobacter vinelandii* N15 using sugar production waste had a weight-average molecular weight (MW) of 1.2 MDa, which is higher than the typical commercial PHB value of around 0.3 MDa. Thermal analysis revealed a melting point of 179 °C and degradation above 275 °C, indicating good thermal stability. Ultraviolet (UV)–visible spectroscopy showed a maximum absorbance at 235 nm. The produced PHB polymer had a purity of 98.9%, a crystallinity of 73%, a density of 1.22 g cm^−3^, a tensile strength of 30 MPa, and an elongation at break of 4.5%, demonstrating a strong but relatively brittle behavior [91]. A comparison of PHB molecular characteristics reported for these bacterial strains is summarized in Table 1.

### 5.2. Thermal, Mechanical, and Physical Properties

PHB-producing microorganisms include a diverse range of bacteria, yeasts, fungi, archaea, cyanobacteria, and other photosynthetic microbes [77]. The physicochemical characteristics of PHB, including its molecular weight, thermal behavior, and mechanical performance, are strongly affected by the microorganism used for its production as well as the cultivation conditions applied during synthesis [92,93]. PHB is a microbially produced biopolymer known for its high crystallinity, biodegradability, and biocompatibility. Unlike chemically synthesized plastics, PHB does not contain catalyst contaminants, which are residual catalyst materials or reaction byproducts that may remain in synthetic polymers after chemical synthesis [94,95,96,97]. PHB also demonstrates good mechanical strength but limited flexibility, with characteristics similar to conventional plastics such as polypropylene and polystyrene [97].

Thermal behavior of PHB can vary depending on the producing microorganism and processing conditions. One study reported that the crystalline regions of PHB began melting at approximately 130 °C and reached complete melting at around 183 °C, with thermal decomposition occurring at about 297.8 °C [17]. In another study, PHB produced by *Azotobacter vinelandii* N-15 showed a melting point of 179 °C and began to degrade only above 275 °C, indicating comparatively good thermal stability for practical applications [97].

Table 2 compares the thermal, mechanical, and physical properties of PHB with selected synthetic and biodegradable polymers [50]. Based on the values presented, PHB highlights several advantages compared with polypropylene (PP). For example, PHB shows about a 13.2% higher tensile strength, a 20% higher crystallinity, and a 36% higher density than PP, indicating better rigidity and structural stability. The melting temperature of PHB is also close to PP, suggesting similar thermal resistance. However, PHB has about a 98.8% lower elongation at break, making it more brittle than PP.

### 5.3. Advantages and Limitations

Advantages: PHB is capable of biodegrading in a broad range of environments, including soil, sludge, freshwater, and marine systems, without the use of specialized composting facilities or chemicals. The material can degrade under both aerobic and anaerobic conditions and break down into harmless byproducts [10,97]. Although PHB degradation can generate secondary microplastic particles, a study conducted under mesophilic anaerobic sludge conditions reported that these particles underwent further microbial degradation [98]. However, the rate and extent of degradation can vary considerably depending on environmental factors such as temperature, moisture, nutrient availability, oxygen availability, and microbial community composition [99]. Consequently, the environmental persistence of PHB-derived microplastics is expected to vary across different environmental conditions [98,99]. Under aerobic conditions, where oxygen is present, PHB mainly breaks down into carbon dioxide, water, biomass, and residual material. In anaerobic environments, where oxygen is absent, degradation produces carbon dioxide, water, methane, biomass, and residue [100], demonstrating the environmental versatility of PHB [10,97]. PHB possesses excellent biocompatibility, allowing it to interact safely with biological tissues and making it suitable for medical use [18]. Moreover, its degradation products, particularly butyrate and 3-hydroxybutyrate, have been associated with improved gut health and potential protective effects against colorectal cancer [101]. Life cycle assessment (LCA) studies have reported that PLA production generates higher carbon emissions and greater ecotoxicity than PHB production. Overall, PHB demonstrates a comparatively lower environmental impact, indicating its strong potential as a sustainable biopolymer for circular economy applications [1]. Due to its high crystallinity, PHB exhibits effective resistance to gas and water transmission, making it a promising material for blending with other polymers in specialized packaging applications [102].

Limitations: Although PHB exhibits a high initial thermal stability, with thermal degradation occurring only at relatively high temperatures, it becomes highly sensitive to overheating near its melting point [80]. As the temperature approaches or exceeds the melting temperature, β-elimination reactions can induce chain scission, resulting in molecular weight reduction and the formation of crotonic acid or crotonate and other volatile degradation products [103]. Furthermore, the degradation temperature of PHB differs from its melting temperature such that degradation begins just above its melting temperature; however, because the processing temperature is close to the melting point, PHB may undergo thermal aging during processing [10]. The processing temperature is very important when PHB is manufactured using compression molding or injection molding, for example. PHB has been reported to exhibit greater thermal sensitivity than conventional petroleum-based plastics due to its narrow processing window and low thermal degradation threshold. As a result, careful control of the melting conditions is required, often alongside the use of stabilizers, plasticizers, or polymer blending strategies to improve processing stability [4,11]. In addition to its thermal stability issues, PHB possesses a high degree of crystallinity (around 60–80%), which exhibits a rigid and brittle behavior with poor flexibility [48,86]. As a result, it has a low elongation at break (approximately 2–15% strain) and a relatively high yield stress exceeding 35 MPa [10]. Furthermore, PHB is synthesized through microbial fermentation and stored as intracellular granules. The process typically produces high-molecular-weight polymer, while obtaining a lower molecular weight requires additional hydrolysis steps, increasing production cost and complexity [10,77].

## 6. Modification Strategies for PHB-Based Materials

PHB blending is widely used to overcome the limitations of pure PHB, such as brittleness, poor thermal stability, high production cost, and limited processing ability. Combining PHB with other biopolymers, fillers, or plasticizers can improve flexibility, mechanical strength, melt processability, and thermal resistance while maintaining its biodegradability and biocompatibility [15,16]. However, the extent of biodegradation depends on the blending material. For example, blending PHB with PLA can reduce the overall biodegradation rate compared with pure PHB, potentially requiring industrial composting conditions for more complete degradation [104]. In contrast, PBS is also biodegradable and can maintain the biodegradable nature of PHB-based blends, although the degradation rate may still be lower than that of pure PHB, particularly in marine environments [105]. These modifications can also enhance barrier and antibacterial properties, making PHB blends in the form of thin films and three-dimensional (3D) printed structures more suitable for sustainable food packaging applications [15,16].

**Figure 6:**
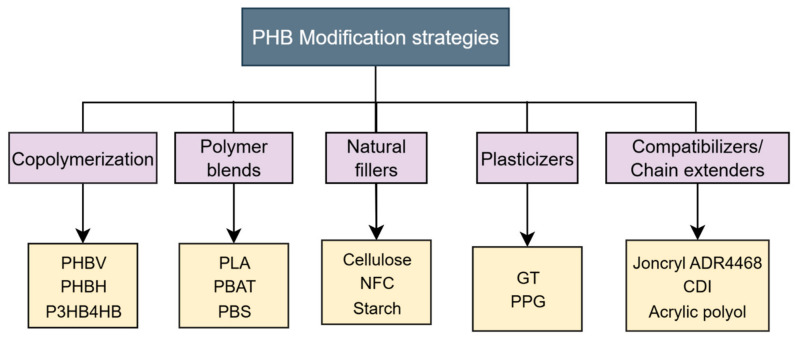
Overview of modification strategies used to improve PHB-based materials. Created by the authors based on the reviewed literature.

Plasticizers and fillers can influence the degradation behavior of PHB-based materials without necessarily reducing their biodegradability. For instance, a study showed phthalate- and glycol-based plasticizers had little effect on PHB degradation, with more than 50% degradation occurring within 24 h and over 98% within 48 h regardless of the presence of additives. While plasticizers primarily improve flexibility, toughness, and processability, certain fillers may enhance biodegradation by increasing water uptake and microbial accessibility, thereby maintaining or even improving the biodegradability of PHB composites [15,106]. Overall, PHB blending offers an effective approach to improve material performance while generally retaining biodegradability, although the degradation behavior depends on the type of blend component, filler, or plasticizer used. The main approaches used to improve the performance of PHB-based materials are summarized in Figure 6. These include copolymerization, polymer blending, incorporation of natural fillers, plasticization, and compatibilizers or chain extenders.

### 6.1. PHB as a Homopolymer

Poly(3-hydroxybutyrate) (PHB) is a homopolymer whose small methyl side groups facilitate close packing of the polymer chains, resulting in the formation of highly ordered lamellar crystalline structures [97]. Under the environmental conditions studied, the PHB homopolymer degraded faster than the PHBV copolymer, indicating that polymer composition can influence microbial biodegradation [107]. In contrast, another study investigated thermal degradation during melt processing rather than environmental biodegradation. That study found PHB was more sensitive to thermal exposure and temperature changes, whereas PHBV and, particularly, PHBH showed greater thermal stability under the investigated processing conditions [80].

### 6.2. PHB Copolymer

The arrangement of two or more distinct monomers within a polymer chain forms a copolymer, and these monomer units may be distributed in random, alternating, or block configurations [108]. PHBV is a bio-based copolymer belonging to the PHA family and is composed of 3-hydroxybutyrate (3HB) and 3-hydroxyvalerate (3HV) monomer units. Compared with the PHB homopolymer, PHBV exhibits improved thermal stability and mechanical performance, making it more suitable for the production of biodegradable materials [109]. Similarly, in comparison to PHB, butyrate units present in poly(3-hydroxybutyrate-co-4-hydroxybutyrate) P3HB4HB, poly(3-hydroxybutyrate-co-3-hydroxyhexanoate) PHBH are composed of hexanoate units that possess longer hydrocarbon side chains [110]. Furthermore, blending PHBH or PHB with amorphous P3HB4HB has been reported to improve elastomeric behavior, particularly when the P3HB4HB content exceeds 30 wt.%, with PHBH/P3HB4HB blends exhibiting the greatest flexibility and elongation among the systems studied [97].

### 6.3. PHB Blends

To enhance material properties, poly(3-hydroxybutyrate) [P(3HB)] in its commercial form has been frequently blended with both natural and synthetic biodegradable polymers. These blends often include bio-based components such as PLA and thermoplastic starch (TPS), as well as biodegradable synthetic polyesters such as PBAT [111]. When blending polyhydroxyalkanoates (PHAs), especially PHB, with other bio-based polymers, additives play a crucial role. For example, plasticizers, nucleating agents, lubricants, chain extenders, and stabilizers have been explored. Careful selection of these additives has helped improve the overall performance of PHB-based composites, hybrids, and blends and ensured better processability, preventing degradation, and minimizing issues like immiscibility [111]. Improving PHB’s properties and lowering production costs can be effectively achieved by blending it with other biodegradable polymers—an affordable strategy that supports its broader industrial adoption [16]. The following studies highlight different PHB blends and strategies to improve their compatibility and performance.

PHB/PBAT/Nanofibrillated Cellulose: In a recent study, PHB and poly (butylene adipate-co-terephthalate) (PBAT), sourced from Biomer (Germany) and BASF (Malaysia), with melting points of 120 °C and 175 °C, respectively, were blended at an 80:20 ratio (PHB:PBAT) to overcome PHB’s inherent brittleness and narrow processing window. The incorporation of PBAT enhanced flexibility, thermal stability, and filament flow in the extruder for use in 3D printers. In another study, nanofibrillated cellulose (NFC), extracted from oil palm trunk fibers via soda pulping, was incorporated into the blend at 0.5 wt.%, 1.0 wt.%, and 2.0 wt.%. Compared with pure PHB, the unfilled PHB:PBAT blend exhibited significant reductions in tensile strength, tensile modulus, and elongation at break by 60.18%, 57.31%, and 37.91%, respectively, while flexural strength and flexural modulus decreased by 25.43% and 19.10%, respectively. In contrast, the impact strength of the PHB:PBAT blend increased by 7.73% relative to pure PHB. With the incorporation of NFC into PHB:PBAT, the mechanical properties of the composites were modified compared with the unfilled PHB:PBAT. For example, at NFC loadings of 0.5 wt.%, 1.0 wt.%, and 2.0 wt.%, tensile strength increased by 96.35%, 104.22%, and 73.63%, respectively, while tensile modulus increased by 18.20%, 25.31%, and 29.52%. Similarly, flexural strength improved by 26.98%, 20.00%, and 8.19%, whereas flexural modulus increased by 20.54%, 5.67%, and 2.93% for the respective NFC loadings. Elongation at break increased by 19.36% and 8.20% at NFC loadings of 0.5 wt.% and 1.0 wt.%, respectively, but decreased by 7.74% at 2.0 wt.% loading, indicating that excessive NFC reduced the ductility of the blend. However, the impact strength decreased by 18.60%, 46.97%, and 45.36% with the addition of 0.5 wt.%, 1.0 wt.%, and 2.0 wt.% NFC, respectively, compared with the unfilled PHB:PBAT blend. Although the 1.0 wt.% NFC composite exhibited the greatest improvement in tensile strength, the 0.5 wt.% NFC formulation provided an overall balanced mechanical performance by simultaneously enhancing tensile, flexural, and ductility-related properties while retaining the highest impact strength among the NFC-filled composites. The authors attributed the deterioration in mechanical performance at higher NFC loadings to particle agglomeration and increased composite stiffness, which restricted polymer chain mobility, reduced stress transfer efficiency, and diminished the material’s ability to absorb impact energy [13].

PHB or PHBV Blended with PLA, PBAT: In another study, PHB and PHBV were blended with PLA and PBAT to improve the 3D filament printability and reduce brittleness. The blends consisted of 70–80 wt.% PHB or PHBV, 15–20 wt.% PLA, and 5–10 wt.% PBAT. The results show PLA enhanced dimensional stability, while PBAT contributed to flexibility and thermal processability. Differential scanning calorimetry showed PHB’s crystallization temperature dropped by around 10 °C when blended with PLA and PBAT, which slowed crystallization and reduced warping. Fourier-transform infrared (FTIR) spectroscopy indicated there was no chemical bonding between PHB and the other polymers because the blend spectra mainly retained the characteristic peaks of the individual polymers, without the appearance of new functional-group peaks or significant peak shifts. This indicates that the polymers were physically blended rather than chemically bonded. Mechanically, PLA slightly reduced PHB’s tensile strength but improved stiffness, while PBAT significantly increased elongation at break and ductility. Among the tested blends, B702505, which contained 70 wt.% PHB, 25 wt.% PLA, and 5 wt.% PBAT, showed the most promising overall performance due to its combined strong mechanical properties, better printability, and reduced warpage [17].

PLA:PHB Blends mixed with HDPE and PP: In this study, each binary polymer combination was prepared at three different weight ratios: 80:20, 50:50, and 20:80, to evaluate biodegradability and FDM 3D printability performance. The blend systems investigated were PLA:PHB, PLA:HDPE, PLA:PP, PHB:HDPE, and PHB:PP. Moreover, the full set of blends included PLA:PHB (80:20, 50:50, 20:80), PLA:HDPE (80:20, 50:50, 20:80), PLA:PP (80:20, 50:50, 20:80), PHB:HDPE (80:20, 50:50, 20:80), and PHB:PP (80:20, 50:50, 20:80). Among the PLA:PHB blends, the 80:20 and 50:50 formulations showed good FDM printability, while the 20:80 blend showed moderate printability. Compared with neat PLA, the PLA:PHB blends exhibited a lower glass transition temperature, indicating increased chain mobility in the amorphous region. This increased chain mobility may have contributed to the enhanced biodegradation behavior observed in these blends. In particular, PLA:PHB (50:50) showed strong biodegradation performance, reaching 85.0% mineralization under controlled composting after 50 days. The improved printability, biodegradation behavior, and tensile modulus of PLA:PHB blends suggest a synergistic interaction between PLA and PHB, likely due to their similar polyester structures [112]. Notably, the 50:50 PLA/PHB blend met ASTM D6400-12 standards for compostability, achieving over 90% biodegradation within 180 days [112].

PBS:PHB and PBS:PLA Blends with Chain Extender: Bio-based polymers including PBS, PLA, and PHB were developed to improve viscosity control, thermal stability, and blend morphology. For example, PBS (BioPBS™ FZ71PB^®^, 50% bio-based) exhibited a melt flow index (MFI) of 22 g/10 min at 190 °C, PLA (Ingeo™ 6201D, 100% bio-based) had an MFI of 15–30 g/10 min at 210 °C, and PHB (ENMAT Y3000P) ranged between 10 and 25 g/10 min at 190 °C. Binary blends of PBS:PHB and PBS:PLA were prepared at 70:30 and 50:50 wt.% ratios, with 0.2 wt.% Carbodilite^®^ (CDI) added as a chain extender to enhance viscosity control and thermal stability. Thermal degradation behavior assessed through thermogravimetric analysis (TGA) revealed a two-step decomposition process, with CDI-containing blends demonstrating enhanced thermal resistance. Differential scanning calorimetry (DSC) analysis indicated an increase in crystallinity over time, particularly in the PLA and PHB phases. Tensile tests showed morphological differences based on blend ratios, where 50:50 blends formed co-continuous phase structures, while 70:30 blends displayed classic matrix-droplet morphologies. Long-term immersion in artificial seawater showed that samples absorbed moisture rapidly within the first two weeks (up to 2.3 wt.%), followed by slower uptake due to hydrolysis and leaching. After six months, PHB-based blends and 3D vertically printed parts showed the most significant reductions in mechanical strength and modulus by as much as 3.3- and 2.5-fold, respectively. Plasticization was not observed on the fractured surfaces, suggesting material embrittlement as the dominant degradation pathway. SEM-EDX confirmed salt crystal accumulation at interlayer junctions, and FTIR indicated only minor chain scission. The authors suggested that future research should assess biodegradation under dynamic conditions involving microbial activity and abrasion [105].

Reactive Compatibilization of PHB/PBAT Blends using Joncryl ADR 4468: In this study, PHB and PBAT were combined at a 70:30 weight ratio to improve the toughness of PHB. Joncryl ADR 4468 was introduced at 1, 2, and 3 wt.% during melt processing, where it acted as both a chain extender and a reactive compatibilizer. The authors also modified cellulose with stearic acid to improve its affinity for the polymer phases before incorporating it into the composite system. The PHB/PBAT blend without Joncryl showed an elongation at break of 22.06% and a toughness of 1.82 MPa. The best mechanical response was obtained with 2 wt.% Joncryl, which increased elongation at break to 85.56% and toughness to 9.40 MPa. At the same time, Young’s modulus decreased from 250 to 177 MPa, indicating greater flexibility. Raising the Joncryl content to 3 wt.% did not produce further improvement and instead lowered the overall performance. The authors therefore identified 2 wt.% Joncryl as the most suitable compatibilizer level for the 70:30 PHB/PBAT blend, while the surface-modified cellulose was further used to improve filler dispersion and reinforcement [113].

### 6.4. PHB with Natural Fillers

Bio-fillers have been incorporated into polymer materials to enhance mechanical performance, lower manufacturing costs, and improve the biodegradability of the resulting composites [114]. These fillers are considered sustainable alternatives to conventional fillers because they are generally renewable, environmentally friendly, biocompatible, and are considered to be low-toxicity materials [115]. These fillers can be derived from different bio-waste sources, including agricultural residues such as corncob waste [116], industrial byproducts such as eggshell waste [117], and marine shell residues [114]. In addition, the incorporation of bio-fillers has been found to alter the crystallization behavior of PHAs, likely due to interactions between the polymer structure, co-monomer composition, and filler particles that influence crystal formation differently [118]. Therefore, the selection of suitable bio-fillers is important for tailoring the properties of PHB-based composites for specific applications.

PHB with Starch Additions: This study examined the use of INDEPEL GUM 90, a modified starch, as a biodegradable additive for PHB. Starch is a natural polymer obtained from plant sources such as cereals and tubers, and it mainly contains amylose and amylopectin. INDEPEL GUM 90 was selected due to its modified form and improved mixing properties with hydrophobic polymers like PHB. PHB/GUM blends were prepared with different starch contents to improve filament quality and reduce the brittleness of neat PHB. Thermal analysis showed that the blends mostly retained the main degradation behavior of PHB, meaning that the starch did not strongly reduce thermal stability. DSC results show that pure PHB had a melting temperature of about 177 °C, while the processed PHB 3D-printable filament showed a lower value of about 167 °C, likely due to heat exposure during extrusion. Blends with lower starch contents, especially 1–10%, showed only small changes in melting behavior. FTIR results confirmed that PHB and starch kept their original structures after extrusion, suggesting no major chemical reaction occurred during blending. SEM analysis showed that lower starch loadings produced a more uniform filament structure, while higher starch contents caused visible starch particle clustering. Overall, small amounts of INDEPEL GUM 90 improved PHB filament quality while maintaining its biodegradable character [119].

PHB with Additions of Microcrystalline Cellulose: In a recent study, microcrystalline cellulose (MCC) was added to PHB in an effort to improve its biodegradability, mechanical performance, and thermal stability. Specimens were fabricated for tensile, flexural, and dynamic mechanical testing. TGA revealed that PHB and its composites degraded in two stages, where an initial weight loss occurred between 250 and 300 °C, followed by further degradation at 300–400 °C. The inclusion of cellulose improved thermal stability by shifting decomposition peaks to higher temperatures, whereas twice-extruded pure PHB (PHB_2) exhibited a 23.89% decrease in tensile strength and an 11.52% decrease in tensile modulus compared with pure PHB due to thermal degradation. When reinforced with MCC at 1.5 wt.% and 3.0 wt.%, tensile modulus increased by 1.82% and 4.24%, respectively, relative to pure PHB. Under flexural loading, PHB_2 showed a 13.50% decrease in flexural modulus compared with pure PHB. With 1.5 wt.% MCC, flexural modulus improved by 7.80% compared with PHB_2, while the 3 wt.% composite showed a minor 0.71% reduction, indicating that higher filler contents did not further enhance stiffness. Tensile strength in the PHB/MCC composites remained 16.67% lower than pure PHB but improved compared with PHB_2. Similarly, flexural strength decreased by 17.24% in PHB_2, by 6.90% in PHB/MCC 1.5 wt.%, and by 13.79% in PHB/MCC 3 wt.% compared with pure PHB. Dynamic mechanical analysis (DMA) showed that the storage modulus (E′) of the MCC-reinforced composites was within 10% of pure PHB and pure PHB_2, suggesting there was minimal reinforcement effect in the viscoelastic region. The glass transition temperature increased from 23 °C for pure PHB to 28 °C for pure PHB_2 and PHB_2/MCC 1.5 wt.% composites and further to 33 °C when 3 wt.% MCC was added to PHB_2, which indicated enhanced thermal resistance and reduced chain mobility at higher filler MCC loadings. However, damping behavior remained unchanged at 1.5 wt.%, as the tan delta (*δ*) curves of PHB_2 and PHB_2/MCC 1.5 wt.% overlapped, confirming limited filler–matrix interaction at lower concentrations. The authors concluded that MCC improved the thermal stability of processed PHB and partly reduced the mechanical losses caused by extrusion and 3D printing. However, weak adhesion between PHB and cellulose limited the reinforcing effect, especially in flexural properties [120].

PHB with Additions of Cellulose and Calcium Carbonate: To improve the functional properties of PHB, natural fillers such as cellulose (CL) and calcium carbonate (CC) were incorporated using a heat-assisted solution casting method. Three formulations were investigated: PHB/CL 90/10, PHB/CC 90/10, and PHB/CL/CC 80/10/10. It was observed that cellulose contributed to smoother film surfaces, while calcium carbonate introduced roughness and visible agglomeration due to filler clustering. Thermal analysis (TGA and DTG) demonstrated that all composites exhibited enhanced thermal stability, with CC-rich blends performing the best. Mechanical tests revealed that dual-filler composites (PHB 80/10/10) had decreased tensile strength and stiffness, likely due to poor dispersion and filler aggregation. While calcium carbonate improved the tensile modulus, the addition of cellulose led to a slight reduction. In laboratory-prepared simulated compost (ISO 20200) with synthetic organic matrix biodegradation tests, all blends fully degraded within eight weeks. Notably, PHB/CL (90/10) achieved complete degradation in 30 days, while PHB/CC (90/10) took up to 50 days, indicating that cellulose enhanced biodegradability, whereas calcium carbonate slowed it down [121].

### 6.5. PHB with Plasticizers

Plasticizers can be bio-based, biodegradable, and synthetic [122]. The use of biodegradable bioplasticizers is a sustainable alternative to synthetic plasticizers due to their low toxicity, good polymer compatibility, and biodegradation. They are commonly derived from renewable sources such as vegetable oils, glycerol, citrates, sugar alcohols, and essential oils. Since PHB is biodegradable, the addition of a biodegradable bioplasticizer creates a fully biodegradable bio-based polymer composite. Plasticizers enhance chain mobility within polymer matrices, promoting structural softening and improving material flexibility. These are generally low-molecular-weight compounds with linear or cyclic carbon structures. When incorporated into the polymer network, they weaken intermolecular interactions and hydrogen bonding, increase free volume, and reduce the energy required for polymer chain movement [123].

PHB with Glycerol Trilevulinate Plasticizers: In a recent study, PHB was blended with non-toxic glycerol trilevulinate (GT), a bio-based plasticizer synthesized via solvent-free esterification of glycerol and levulinic acid to enhance its melt-processability and mechanical performance. PHB compounds were prepared with 2.5, 5, and 10 wt.% GT using a twin-screw extruder. Injection molding was used to create the tensile dog-bone-shaped test specimens, which were then assessed for their mechanical, thermal, and rheological behavior. The addition of GT led to a significant reduction in the glass transition temperature, which dropped from 4 °C for neat PHB to −5 °C when 5 wt.% GT was added, indicating effective plasticization. Melting temperature and crystallinity were also reduced, which improved the material’s flexibility. At 5 wt.% GT, the tensile modulus decreased by 28%, while toughness increased by approximately 10%. Rheological analysis showed shear-thinning behavior and reduced melt viscosity with increasing GT content. The zero-shear viscosity (*η*_0_) of pure PHB was recorded at 65 Pa·s. As the plasticizer content increased, the viscosity of the material decreased proportionally. Compared with pure PHB, the addition of GT resulted in a reduction of 18.5% at 2.5 wt.%, 27.7% at 5 wt.%, and 46.2% at 10 wt.% plasticizer content. Overall, the authors concluded that 5 wt.% GT offered the best balance between flexibility, reduced melt viscosity, and improved processability, making GT a promising sustainable plasticizer for PHB [124].

### 6.6. PHB with Fillers and Plasticizers

Both blending with other biodegradable polymers and the addition of fillers to PHB are effective strategies used to modify and improve the properties of PHB. This method can tailor the final PHB material to be competitive with conventional plastics in packaging and in the development of biodegradable 3D-printable filaments. For PHB to be more competitive, PHB properties and production costs can be improved by blending and careful selection of filler types and amounts. PHB interaction with plasticizers and fillers can lead to properties with mixed effects, sometimes reducing thermal and mechanical properties [16].

PHB/PLA/Plasticizer/Hydroxyapatite Composite: To improve the processability and flexibility of PHB in biocomposites for 3D-printable filament applications, polypropylene glycol (PPG) was incorporated as a plasticizer into a fixed blend of 70 wt.% PHB and 30 wt.% PLA. The optimal PPG concentration for workable melt flow characteristics was identified as 9.1 wt.%. Hydroxyapatite (HA), synthesized via wet precipitation, was added at varying loadings from 9.1 to 27.3 wt.% in an effort to reinforce the composites. The addition of PPG reduced the viscosity of the blend and improved polymer chain mobility, resulting in increased flexibility and a noticeable decrease in the glass transition, melting, and crystallization temperatures. The addition of HA disrupted PHB’s crystallinity, as confirmed by X-ray diffraction (XRD), with higher HA contents leading to a lower melting enthalpy. At 10 wt.% HA loading, the flexural modulus improved, but excessive HA caused particle agglomeration and reduced impact strength due to poor interfacial adhesion. Despite a slight drop in flexural properties compared with PLA, the elongation at break of the plasticized PHB/PLA/HA composites was about 50% higher than commercial PLA, indicating enhanced toughness. These results confirm that incorporating PPG and moderate HA contents offers a practical solution for developing biodegradable filaments with balanced mechanical properties suitable for sustainable 3D printing [125].

Overall, these studies show that neat PHB often needs to be blended with other polymers or modified with fillers, plasticizers, chain extenders, or compatibilizers to reduce brittleness, improve melt processing, and enhance mechanical performance. Compatibilizers can improve adhesion between immiscible polymer phases, leading to better mechanical performance. However, the final properties depend on the type and amount of additive used, since excessive loading can lead to agglomeration, poor dispersion, weak interfacial bonding, and lower toughness. Therefore, careful formulation and well-optimized PHB-based blends have strong potential for biodegradable packaging, biomedical applications, and 3D-printable filaments.

### 6.7. Conventional Processing of PHB-Based Materials

Extrusion and injection molding provide practical processing routes for producing PHB-based materials. However, the broader industrial application of PHB remains challenging because its high crystallinity and sensitivity to heat, oxygen, and mechanical shear which can promote degradation during melt processing. This degradation reduces molecular weight and can adversely affect mechanical performance. Consequently, additives are often incorporated to improve the processing stability and overall properties of PHB [126]. Chain extenders are particularly useful because they can compensate for processing-induced chain scission by increasing molecular weight and melt viscosity, thereby improving melt stability and material performance [127].

Reactive Extrusion: Reactive extrusion combines melt compounding with chemical modification in a continuous processing step. In a related study, P3HB was processed by twin-screw reactive extrusion using 0.5 wt.% dicumyl peroxide and 1–10 wt.% acrylic polyol, followed by injection molding. Acrylic polyol improved the molecular weight, melt viscosity, thermal stability, and processability of P3HB, with the 5 wt.% formulation providing the best overall balance of properties. The findings indicate that reactive extrusion can enhance the processing stability of P3HB and offers potential for continuous manufacturing using conventional polymer-processing routes [128].

Injection Molding: Injection molding is widely used to shape thermoplastic materials into reproducible components. In a previous work, PHB/TPS blends containing oat or corn cob fibers were first compounded by extrusion, and then the materials were injection molded into plates. Before molding, the pellets were dried to reduce moisture-related processing defects, and the blends were processed under controlled barrel temperature, mold temperature, and pressure conditions. The study demonstrates that PHB-based blends and natural fiber composites can be shaped using established thermoplastic processing equipment, supporting the potential application of injection molding in the production of biodegradable components [129].

While extrusion and injection molding remain suitable for continuous and high-volume production, additive manufacturing provides an alternative route for fabricating customized PHB-based components with complex geometries. Therefore, the choice of processing method depends on production scale, component design, material formulation, and final application [9].

## 7. Additive Manufacturing of PHB

### 7.1. Overview of Additive Manufacturing

Additive Manufacturing (AM), as defined by ISO/ASTM 52900:2021, involves building components layer-by-layer from 3D digital models, distinguishing it from traditional subtractive and formative methods. The standard outlines seven AM process types: binder jetting, direct energy deposition, material extrusion, material jetting, powder bed fusion, sheet lamination, and vat photopolymerization [130]. Initially known as rapid prototyping, AM streamlined computer-aided design (CAD)-to-part fabrication, enhanced design communication, and reduced time and costs [131]. Evolving beyond prototyping, it now supports full-scale manufacturing across aerospace, automotive, biomedical, and consumer sectors using materials ranging from polymers to composites [132,133]. Material roles include build and support functions and processes such as fused filament fabrication (FFF), which are now able to accommodate composites [132]. Despite challenges of low mechanical strength in polymers [13], polymer-based techniques including stereolithography, inkjet printing, selective laser sintering, and fused deposition modeling remain prevalent due to versatility and ease of processing [14,133]. AM also holds promise for sustainability by reducing waste and emissions through effective adoption [134,135].

### 7.2. FDM/FFF Processing of PHB

FDM, also known as FFF, was developed by Scott Crump in 1988 and is the most widely used polymer-based AM method [14,135,136]. It builds structures by extruding heated thermoplastic filament through a nozzle in layers. Its accessibility and cost-effectiveness make it particularly dominant in the 3D printing industry [136]. Recently, biopolymers derived from plants and microorganisms have been gaining attention in FDM for their biodegradability, biocompatibility, and low toxicity, which is ideal for sustainable applications like wound healing and drug delivery [119,135].

### 7.3. Challenges in FDM 3D Printing

Highly crystalline polymers such as PHB often warp during 3D printing due to rapid shrinkage from crystallization. Controlling the cooling rate is difficult as PHB continues to crystallize quickly even after processing at room temperature [9]. PHB’s natural drawbacks, such as limited thermal stability, brittleness from its inherent high crystallinity, and narrow processing range, make it difficult to process and manufacture [122]. Overcoming these challenges is essential to improve the performance and reliability of PHB in 3D printing [13].

To mitigate PHB’s limitations, researchers have explored advanced processing methods and composite blends to improve its performance [9]. In this review, the influence of key generic printing parameters on the mechanical behavior of pure bio-sourced PHA and its blends was examined within the context of material extrusion (MEX) additive manufacturing.

### 7.4. Effect of Processing Parameters

Mechanical performance in MEX 3D printing is highly sensitive to factors such as nozzle temperature, print speed, layer thickness, and strand width [17]. These factors influence bonding between layers, porosity, warping, and overall part strength [137].

Among these, layer thickness has the most significant effect on tensile properties, where a higher thickness improves tensile strength but reduces impact resistance. Nozzle temperature also plays a key role, especially in enhancing interlayer adhesion and impact strength. Print speed tends to reduce both tensile and impact strengths when increased, though its effects depend on the specific polymer type used. Strand width can improve bonding and tensile strength, but excessive width may lead to printing defects like over-extrusion or poor dimensional accuracy. For PHAs and similar biodegradable polymers, fine-tuning these parameters can greatly improve mechanical performance [138]. When preparing PHA for extrusion, to prevent hydrolysis and maintain the integrity of the PHA materials, it has been reported in the literature to vacuum-dry the PHA pellets prior to processing. Careful control of temperature and screw speed during extrusion was essential to avoid thermal degradation, overheating, and material burning [139].

### 7.5. PHB-Based Composite Filaments for FDM

Similar to PHA, PHB faces several processing limitations, primarily due to its narrow thermal window, as its melting point lies close to its degradation temperature. In addition, its high crystallinity results in brittleness and low elongation at break. Also, pure PHB is challenging to process through 3D printing, as excessive warpage during fabrication can lead to poor dimensional accuracy and part defects [140].

Although PHB exhibits limited thermal stability during melt processing, it remains an attractive biodegradable polymer for selected applications due to its favorable mechanical performance after processing. Consequently, numerous modification strategies have been developed to improve its processing characteristics while preserving its desirable end-use properties [131,132].

To overcome its processing challenges and broaden its application potential, various modification strategies have been explored in the literature. These include copolymerization, side-chain functionalization, polymer blending, and the incorporation of additives such as plasticizers, nucleating agents, and lubricants. Such enhancements not only improve PHB’s processability and mechanical behavior but also support its integration into diverse fields, including biomedical, pharmaceutical, food packaging, agricultural, and environmental applications [14]. Certain fillers also function as nucleating agents, improving PHB’s crystallization by promoting the formation of smaller, more uniform crystals. This leads to a higher crystallization temperature (*T*_c_) and faster crystallization rates. Additives such as boron nitride, talc, titanium dioxide (TiO_2_), and chitin nanocrystals have been added to PHB to produce 3D-printable filaments and have been shown to enhance the crystal structure, reduce brittleness, and improve mechanical performance [134].

For manufacturing 3D-printable filaments, PHB has been blended with secondary biopolymers, as well as the addition of various fillers to improve processability, printability, and mechanical performance, as outlined in Table 3. Examples include, PHB or PHBV with PLA/PBAT [17], PHB/PBAT/NFC [13], PBS/PHB and PBS/PLA blends with Carbodilite^®^ HMV-15CA (CDI) [105], PHB/PLA/corncob [116], PHB/Starch filaments [119], PHB blends—filler (cellulose) [120], PHB/GT [124], PHB/PLA/HA [125] and PLA/PHB/HDPE/PP [141].

As shown in Table 3, most PHB-based filaments were processed within a nozzle temperature range of approximately 170–210 °C, depending on the blend composition and additive type. Bed temperature varied from ambient conditions to 60 °C. Filament diameters were commonly prepared around 1.75 mm or 2.85 mm, which are standard sizes for FDM/FFF printing. The variation in processing conditions indicates that PHB-based materials require careful parameter optimization because their printability strongly depends on blend composition, filler loading, and thermal stability.

PHB, PHBV, and PLA, PBT Blended Filaments: PHB- and PHBV-based biodegradable filaments were prepared for FDM 3D printing to improve the poor printability of neat PHB and PHBV. Pure PHB and PHBV were difficult to process because their filaments showed breakage during winding, and the printed parts were affected by warpage. In this study, PHB or PHBV was used as the main polymer phase at 70–80 wt.%, together with PLA and PBAT as supporting components to improve filament processing and printing performance. Before extrusion, the polymer pellets were dried at 70 °C for 2 h. The blends were then melt-compounded using a twin-screw extruder at 170 °C, and the screw speed was controlled between 30 and 50 rpm to produce filaments with a diameter of 1.6–1.8 mm. The printed specimens were fabricated using a 0.4 mm nozzle, 190 °C nozzle temperature, 30 °C bed temperature, 40 mm/s print speed, 0.2 mm layer height, and 100% infill. Blending improved filament stability and reduced printing defects compared with neat PHB and PHBV. Among the tested formulations, B702505, containing 70 wt.% PHB, 25 wt.% PLA, and 5 wt.% PBAT, and V702505, containing 70 wt.% PHBV, 25 wt.% PLA, and 5 wt.% PBAT, showed the best overall printing performance, with better dimensional stability, lower warpage, and improved surface quality. Overall, the study showed that PHB- and PHBV-rich blends can be processed into promising biodegradable filaments for sustainable FDM 3D printing [17].

PBS/PHB and PBS/PLA Blended Filaments with a Chain Extender: PBS/PHB and PBS/PLA blends containing a chain extender, Carbodilite^®^, were also processed for fused filament fabrication (FFF) applications. Blends were compounded using a twin-screw extruder operating between 175 and 190 °C, and filaments of 1.75 mm diameter were produced. Samples were printed using FFF with a 0.4 mm nozzle, a print speed of 40 mm/s, and a layer height of 0.2 mm. SEM analysis identified microvoids and structural irregularities, especially in vertically printed specimens, and crystallite growth was evident at interlayer boundaries. The results demonstrate that blended compositions and printing orientations strongly influenced the structural integrity of printed parts. After six months of immersion in static artificial seawater, the printed samples absorbed water and showed signs of salt deposition, hydrolytic aging, and loss of mechanical strength. However, the degradation of the bulk-printed parts was slow, and the samples generally retained their structure. The study indicates that PBS/PLA blends offered better mechanical durability, whereas PBS/PHB blends, particularly the 50/50 composition, were more prone to degradation. Horizontally printed samples also showed better stability than vertically printed ones, mainly because they had fewer defects and absorbed less water [105].

PLA and PHB Blended with HDPE and PP Filaments: PLA and PHB blends were processed under the same conditions using a single-screw extruder with a temperature profile ranging from 170 to 190 °C and a screw speed of 3.5 rpm. The filaments were extruded to a diameter of 1.75 mm, monitored using Arduino software, and stored at 40 °C with silica gel until further testing. Only neat PLA and PLA-based blends were suitable for 3D printing and tensile testing. PHB and its blends with HDPE or PP could not be printed due to processing challenges, particularly poor melt flow and filament breakage. Given the lack of dedicated recycling for 3D printing waste, using fully biodegradable material and industrially compostable blends [104] could help address environmental concerns [141].

PHB/PBAT/Nanofibrillated Cellulose Composite Filaments: PHB/PBAT/NFC filaments were prepared for FDM 3D printing to improve the limited printability of neat PHB. PBAT was added at an 80:20 (PHB: PBAT) weight ratio to improve melt flow, flexibility, filament stability, and layer adhesion. The composites were extruded into filaments at 180–190 °C with a screw speed of 45–50 rpm, and the filament diameter was kept between 2.85 and 3.00 mm. Printing was carried out using 100% infill, 60 mm/min speed, 60 °C bed temperature, and 210 °C nozzle temperature. The PHB/PBAT blend printed more smoothly than pure PHB, while 0.5 wt.% NFC improved surface quality and interlayer bonding. Higher NFC loadings caused rough surfaces, filler agglomeration, and possible nozzle blockage. Overall, 0.5 wt.% NFC was considered the best loading for balancing printability, filament quality, mechanical performance, and thermal stability [13].

PHB/PLA/Corncob Composite Filaments: In this study, PHB and PLA were blended at 55:45 wt.% and used as the main material for FFF 3D printing. Corncob powder was added at 2, 4, 6, and 8 wt.% to study how the filler affected printing behavior. The composite filaments were made using a Filabot single-screw extruder with a target diameter of 2.85 mm. Before printing, the filaments were dried at 50 °C for 12 h to remove moisture. Printing was performed using an Ultimaker 3 printer at 200 °C. The authors selected this temperature because printing at lower temperatures caused nozzle blockage and visible defects in the printed parts. The PHB/PLA blend printed well without corncob, while the addition of corncob changed the flow behavior depending on the filler amount. Lower and moderate filler contents were easier to print, but 8 wt.% corncob caused more particle clustering and reduced performance. Overall, the study showed that PHB/PLA with up to 6 wt.% corncob was more suitable for FFF printing than blends with higher filler loading [116].

PHB/Starch Composite Filaments: PHB/starch composite filaments were prepared for FDM 3D printing using a custom-built single-screw extruder. Before extrusion, PHB and INDEPEL GUM 90 were mixed at 1500 rpm for 30 s. Among the tested extrusion settings, 170 °C and 20 rpm produced the most suitable filament, with a diameter of about 1.74 ± 0.10 mm. The PHB/GUM 5 (95% PHB and 5% INDEPEL GUM 90) filament was selected for printing because it had a suitable diameter and better filament quality. Printing was carried out using a Boa 3D Stella FDM printer at 170 °C nozzle temperature, 15 mm/s print speed, 20% infill, and an unheated bed at 25 °C. The PHB/GUM 5 filament was successfully printed into a scaffold with a diameter of 10.75 mm and a thickness of 5 mm. Microscopy images showed connected printed strands, good layer joining, and small surface pores. The study also reported that pure PHB did not print successfully, while lower starch contents, especially around 5%, improved printability. Overall, PHB/GUM 5 showed promise as a biodegradable filament for FDM-printed scaffold structures [119].

PHB Microcrystalline Cellulose Composite Filaments: PHB-based composite filaments were prepared for FDM by incorporating microcrystalline cellulose (MCC) into the PHB matrix. In this study, the authors first produced a masterbatch containing 15 wt.% cellulose, which was later diluted with pure PHB to obtain final MCC contents of 1.5 wt.% and 3 wt.%. Prior to processing, the PHB pellets and MCC powder were dried at 70 °C for 12 h to remove moisture and reduce hydrolytic degradation during melt processing. The materials were compounded using a co-rotating twin-screw extruder with a temperature range of 155–160 °C and a screw speed of 150 rpm. The compounded materials were then converted into 2.85 mm filaments using a desktop filament extruder. The filaments were printed with an Ultimaker 3D printer using a nozzle temperature of 185 °C, a 0.1 mm layer thickness, a 45° raster angle, a 100% infill, and an unheated build platform. Dog-bone and rectangular specimens were printed for tensile, flexural, and dynamic mechanical testing. Overall, the study demonstrated that PHB/MCC composites could be processed into printable biodegradable filaments for FDM applications, showing the potential of cellulose-filled PHB materials for sustainable 3D-printed parts [120].

PHB Filaments with a Plasticizer: PHB filaments were prepared for FDM 3D printing by adding glycerol trilevulinate (GT), a bio-based plasticizer, to improve the melt processing behavior of PHB. PHB was first blended with 2.5, 5, and 10 wt.% GT using a twin-screw extruder, and further converted into printable filaments through a single-screw extruder. The filament diameter was controlled at 1.55 ± 0.1 mm. Porous cylindrical scaffolds with a diameter of 15 mm and a height of 5 mm were printed using a Prusa i3 MK3S+ printer. The printing parameters included a 0.4 mm nozzle, a 0.2 mm layer height, a 0.4 mm strand width, a 30% linear infill, a 25 mm/min printing speed, and a 60 °C build plate. The infill direction was changed by 90° between layers, and the scaffolds were printed with two wall loops, no top or bottom layers, brim support, and glue for bed adhesion. Printing temperatures from 180 to 200 °C were evaluated. The results show that GT improved PHB printability and allowed the processing temperature to be reduced. Among the tested formulations, PHB with 5 wt.% GT showed the best printing performance at 180 °C, producing smoother, more continuous strands and a regular porous scaffold structure. Compared with commercial plasticizers such as acetyl tributyl citrate (ATBC) and 1,2-cyclohexane dicarboxylic acid diisononyl ester (DINCH), GT has better scaffold quality by forming more even strands and reducing visible defects between printed layers. Overall, the study showed that GT made PHB easier to print by FDM and allowed porous scaffold structures to be produced for possible biomedical uses [124].

PHB/PLA/Plasticizer/Hydroxyapatite Composite Filaments: PHB/PLA/HA bio-composite filaments were produced for FFF 3D printing by combining PHB and PLA with polypropylene glycol (PPG) and hydroxyapatite (HA). The base polymer blend contained 70 wt.% PHB and 30 wt.% PLA, while 9.1 wt.% PPG was used to make the material easier to extrude during filament making and printing. HA was added at 9.1, 18.2, and 27.3 wt.% to prepare different composite filaments. The mixtures were processed in a twin-screw extruder at 170 °C with a screw speed of 40–50 rpm, producing filaments with a diameter of 1.70–1.85 mm. The printed samples were made using a WANHAO Duplicator 6 printer with a 0.4 mm nozzle, a 210 °C nozzle temperature, a 40 °C bed temperature, a 0.2 mm layer height, a 100% infill, and a 25 mm/s print speed. Among the prepared materials, the formulation with 9.1 wt.% HA and 9.1 wt.% PPG showed the smoothest material delivery through the nozzle and produced the most reliable printed specimens. When the HA amount was increased, the material became more difficult to print because the filler particles limited melt movement and created particle clustering. Overall, the study showed that PPG-plasticized PHB/PLA/HA filaments can be used for FFF 3D printing, with the 9.1 wt.% HA and 9.1 wt.% PPG formulation giving the most suitable printing performance [125].

Overall, these studies show that neat PHB is difficult to print because of its brittleness, low melt flow, and narrow processing window. Blending PHB with other polymers or adding plasticizers and fillers can improve filament formation, printing stability, and the quality of printed parts. Therefore, modified PHB-based materials show good potential for sustainable FDM/FFF 3D printing, particularly for biodegradable packaging and biomedical scaffold applications.

## 8. Biodegradation of PHB

### 8.1. Biodegradation Mechanism

**Figure 7:**
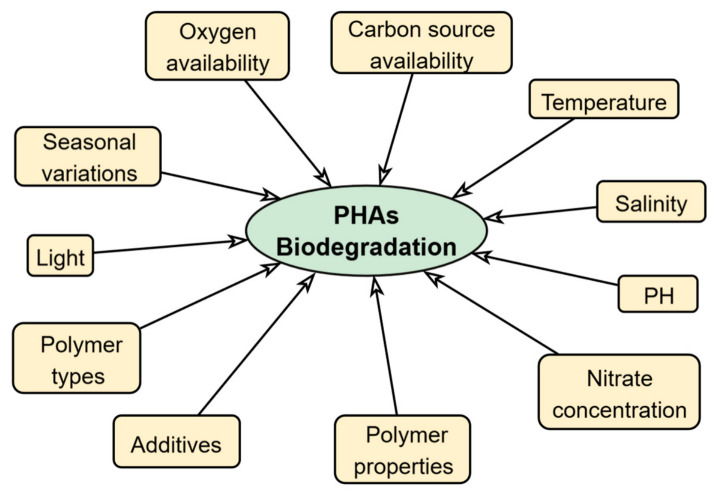
Factors affecting the microbial biodegradation of PHAs drawn by the authors using data available in [107].

PHA and PHB biopolymers are robust and behave similarly to conventional plastics. For example, their mechanical properties such as tensile strength, tensile modulus, and elongation at break are comparable to synthetic plastics, but once exposed to soil, marine, and freshwater environments, they will fully degrade [10]. Biopolymer degradation by enzymes involves two steps: enzyme binding to the polymer, followed by hydrolytic breakdown. The slowest phase is microbial attachment and colonization on the plastic surface. Biodegradable polymers like PHA and PHB are broken down into small molecules and eventually mineralized into nontoxic byproducts such as carbon dioxide (CO_2_) and water (H_2_O) [50,142]. The breakdown of polymers by biological processes depends on several key factors, as summarized in Figure 7. Along with the chemical structure of the polymer itself, the type of microorganism present and environmental conditions such as temperature, PH, and nutrient availability can significantly affect how quickly degradation occurs [107,143].

### 8.2. Performance in Environments

Biodegradation is the complete breakdown of carbon-based plastics by naturally occurring microbes into carbon dioxide, water, and biomass, without leaving behind microplastic residues. A recent study tested three biodegradable plastics—PHB, polybutylene sebacate (PBSe), and polybutylene sebacate co-butylene terephthalate (PBSeT) alongside conventional low-density polyethylene (LDPE) in marine environments using lab, mesocosm, and field tests across different environments such as beach, seafloor, and open water habitats. PHB showed the fastest and most consistent degradation, with its half-life ranging from just 54 days on the tropical seafloor to 1247 days in cold open water. In contrast, LDPE showed no degradation. Environmental conditions had a strong influence on degradation rates, with PHB breaking down more rapidly on the seafloor, while PBSe and PBSeT performed better on beaches. No significant breakdown occurred in open water for any material, highlighting that biodegradability depends heavily on location and conditions [144].

PHB is capable of breaking down in both oxygen-rich (aerobic) and oxygen-poor (anaerobic) environments, making it environmentally friendly and eliminating the need for specific conditions to degrade effectively [145]. In a related study, the degradation of the PHB homopolymer required approximately 250–270 days. In a tropical monsoon environment, such as Hoa Lac soil, the PHBV copolymer film still retained about 40% of its mass after 360 days. Different soil conditions also influenced degradation behavior, where slightly sandy or loamy soil showed about 40% degradation of PHB films after 360 days, while PHBV films degraded by roughly 25% over the same period [146].

In a study conducted under oxygen-rich (aerobic) conditions in water, three bioplastics—PHB, PHBV, and PCL—demonstrated substantial biodegradation over 117 days. Final biodegradation levels, measured as CO_2_ conversion, reached 83.0% for PHB, 87.4% for PHBV, and 77.6% for PCL. These results suggest that all three materials either meet or come very close to meeting the biodegradability requirements of the International Organization for Standardization (ISO) 14852, which requires approximately 90% degradation within 90 days when compared with a positive control like cellulose. Interestingly, all three materials showed an initial lag of about 11–13 days before degradation began. This delay was likely due to the time needed for microorganisms in the activated sludge to adapt and start producing the necessary enzymes to break down the bioplastics. After this adaptation period, the materials degraded rapidly, especially up to 45 days. PCL degraded slightly faster than the others during this phase, but still not as quickly as cellulose, which reached 86.8% biodegradation in just 68 days. In contrast, other tested materials—PBS, PBAT, PLA, and a PLA-PCL blend showed little to no signs of degradation under the same conditions, indicating they are not biodegradable in aqueous aerobic environments. HDPE was used as a negative control, which also showed no carbon dioxide release or oxygen consumption, confirming the validity of the test setup according to ISO 14852 guidelines. When tested in an oxygen-free (anaerobic) water-based environment, PHB and PHBV broke down significantly, reaching degradations of around 84% and 81%, respectively, in just 77 days. This was based on the amount of carbon dioxide, methane, and dissolved carbon released. On the other hand, plastics such as PBS, PBAT, PLA, and the PLA/PCL blend barely degraded at all, with less than 5% breakdown, confirming the reliability of the test under ISO 14853 guidelines [147]. However, it was observed that in an anaerobic water-based condition, PHB showed higher degradation than PHBV.

### 8.3. Enhancement of PHB Biodegradation

The degradation of PHA polymers depends on several environmental and material-related factors. Conditions such as oxygen availability, temperature, light, pH, salinity, nitrate concentration, and seasonal changes can affect how quickly degradation occurs. Polymer-related factors, including polymer type, molecular structure, additives, and the availability of other carbon sources, can also influence the degradation rate [107]. Under aerobic conditions, PHA polymers are mainly broken down into carbon dioxide and water. In contrast, anaerobic conditions support microbial digestion, which can lead to methane and carbon dioxide production. Previous studies have shown that anaerobic digestion can generate measurable methane yields; however, the degradation process may be slower than in composting environments. The presence of nitrate also accelerated degradation by enhancing the growth and activity of denitrifying microbes across varying aeration conditions [92]. Additives such as bioplasticizers improve the flexibility and strength of PHB, but they may also influence its biodegradation by affecting microbial activity [107]. In another study, a low-pressure plasma treatment was used to modify the surface of PHB, making it more hydrophilic and increasing its surface roughness. These changes improved microbial adhesion and biofilm formation, leading to a 1.5-fold faster biodegradation in soil. This method showed promise for enhancing the degradation of PHB and other biodegradable polymers at the end of their life [145].

## 9. Applications and Market Adoption

In terms of current applications, due to their biodegradability, PHB and PHAs are explored for packaging, single-use plastic products, and compostable films, coatings, and biomedical uses such as sutures, tissue scaffolds, and drug delivery [19]. Since PHB is obtained from renewable resources, as well as being biocompatible and able to biodegrade into non-toxic products, it is of high research interest where plastic pollution and fossil resource dependence are considered.

### 9.1. Packaging and Food Industry Applications

PHB is regarded a promising material for packaging and 3D printing due to its high mechanical stability, creep resistance, thermal stability during long-term storage, and strong resistance to UV radiation [16]. The biodegradable and biocompatible characteristics of PHB make it a highly attractive candidate for sustainable packaging applications, supporting the commercialization of compostable products such as shopping bags, food containers, drinking straws, single-serve coffee pods, mulching films, and paper coatings [19,74]. In food packaging applications, barrier properties are important for limiting the transmission of oxygen and moisture, thereby helping to preserve food quality and extend shelf life. PHAs exhibit moderate barrier performance and allow the permeation of small molecules such as oxygen, carbon dioxide, water vapor, organic vapors, and certain liquids [10]. A controllable biodegradation strategy for PHB films was developed using a cellulose triacetate (CTA) coating, which temporarily delayed degradation and could be reactivated under alkaline or composting conditions. This environmentally responsive approach may be useful for applications such as marine-disposable products, agricultural films, and smart packaging materials [134].

### 9.2. Biomedical and Pharmaceutical Applications

Additive manufacturing has expanded the use of PHB and its blends in biomedical applications by enabling the fabrication of biocompatible structures such as scaffolds, stents, sutures, and meshes [10]. The biomedical sector is an emerging application area for PHB due to its FDA (U.S. Food and Drug Administration) approved biosafety for medical use, including its application in TephaFLEX absorbable sutures [148]. Due to its biocompatible nature, PHB has been explored for applications such as wound healing, tissue engineering, drug delivery devices [149], dental restorative materials, and dental pulp-capping procedures [18].

### 9.3. Agricultural Applications

PHB-based materials have also been investigated for biodegradable mulch films and controlled-release agricultural systems [150]. PHAs are promising biodegradable coating materials for slow-release fertilizers, with the potential to improve nutrient use efficiency, enhance crop productivity, and reduce environmental pollution [151].

### 9.4. Commercial PHB Products and Industrial Players

Several global companies and industrial players manufacture or develop PHB on a commercial scale, which are listed in Table 4.

## 10. Challenges and Research Gaps

Although PHB has many environmental advantages, several technical and economic limitations still restrict its large-scale industrial implementation. One of the major challenges associated with PHB is its inherent brittleness and low elongation at break, which are mainly caused by its high crystallinity and rigid molecular structure [10,12]. These properties reduce flexibility and limit its application in products requiring high toughness or impact resistance. In addition, PHB exhibits a narrow thermal processing window because its melting temperature lies close to its degradation temperature, increasing the risk of thermal degradation during extrusion, injection molding, and additive manufacturing processes [4,11]. Another major limitation is the current high production cost of PHB. Since PHB is produced through microbial fermentation, overall manufacturing costs are strongly influenced by feedstock selection, microbial productivity, fermentation efficiency, and downstream purification requirements [84,85]. Although renewable waste-derived feedstocks such as crude glycerol, agricultural residues, and industrial byproducts have shown promising cost-reduction potential, their large-scale integration remains limited [85]. From a materials engineering perspective, blending PHB with other biodegradable polymers, fillers, nanofillers, and plasticizers has significantly improved toughness, thermal resistance, and processability [13,111,124,152]. However, issues such as poor interfacial compatibility, filler agglomeration, phase separation, and reduced toughness at excessive filler loadings remain unresolved [16,153]. In additive manufacturing, PHB-based filaments show strong potential for FDM/FFF applications due to their biodegradability and dimensional stability. Nevertheless, poor melt flow, warping, thermal shrinkage, interlayer defects, and limited printability still restrict broader adoption [9,13,138,152]. In terms of environmental performance, PHB demonstrates effective biodegradation under soil, marine, freshwater, composting, aerobic, and anaerobic conditions [144,145,146,147]. However, degradation rates vary significantly depending on environmental factors such as temperature, microbial activity, moisture, oxygen availability, and polymer composition [107,144]. Based on the current literature, several important gaps remain in PHB production, processing, biodegradation, and commercialization. Improving microbial strains, renewable waste-derived feedstocks, and fermentation conditions such as nutrient limitation, oxygen transfer, mixing, feeding strategy, and continuous cultivation could help increase PHB productivity and support industrial scale-up [10,11,74,75,139]. Greener extraction and purification methods are also needed to reduce solvent use, energy demand, and polymer degradation while maintaining high recovery efficiency [11,74,75]. In PHB blends and composites, greater attention should be given to blend composition, filler loading, compatibilization, and processing conditions to achieve a better balance among mechanical performance, thermal stability, barrier properties, and biodegradability [12,17,75,118]. For additive manufacturing, the effects of nozzle temperature, print speed, layer height, raster orientation, infill density, and cooling conditions should be examined more systematically in relation to print quality, dimensional stability, and long-term durability [17,105,125]. Further work is also required to clarify how crystallinity, crystal morphology [10], molecular-weight reduction, and processing history influence degradation and thermo-mechanical behavior [118]. Standardized biodegradation studies across soil, compost, freshwater, marine, aerobic, and anaerobic environments would improve understanding of how temperature, moisture, oxygen availability, microbial activity, and material characteristics affect PHB degradation. Finally, integrating techno-economic and life-cycle assessments with process development would help evaluate commercial feasibility and identify the main technical, economic, and environmental barriers to large-scale PHB use [74].

## 11. Future Outlook

Increasing environmental concerns, together with growing restrictions on selected single-use plastic products, have intensified the search for sustainable alternatives to conventional fossil-based polymers. As highlighted throughout this review, PHB offers considerable potential as a renewable and biodegradable material for a wide range of applications. Building on the challenges and research gaps discussed in the previous section, further progress in PHB will require improvements across the entire production and application chain. Efforts should focus not only on increasing production efficiency and enhancing material properties, but also on developing better biotechnological tools, more reliable processing methods, and practical strategies for industrial scale-up. Future studies should therefore take a more integrated approach by combining renewable feedstocks, microbial and metabolic engineering, advanced material design, efficient manufacturing technologies, and life-cycle assessment. Progress in these areas could help reduce production costs, improve the performance and processability of PHB, and support its wider use in packaging, biomedical products, agriculture, and engineering applications.

### 11.1. Sustainable Feedstocks

Future PHB production is expected to increasingly rely on low-cost and renewable feedstocks, particularly agro-industrial residues, lignocellulosic biomass, and other residual carbon sources [154].

In addition, integrated biorefinery approaches that couple biomass valorization with the production of PHB and other value-added products represent a promising strategy for maximizing resource efficiency and improving process economics. Such integrated systems can reduce waste generation, increase biomass utilization, and contribute to the development of more sustainable and commercially viable PHB production processes [155].

### 11.2. Genetic and Metabolic Engineering

Advances in metabolic engineering, synthetic biology, and strain optimization are expected to improve microbial productivity, substrate utilization efficiency, and polymer quality. Advances in metabolic engineering, systems biology, synthetic biology, and strain optimization are expected to further improve microbial productivity, substrate utilization efficiency, and polymer quality. Emerging systems metabolic engineering approaches that integrate computational modeling, adaptive laboratory evolution, and Design–Build–Test–Learn (DBTL) strategies may accelerate the development of robust microbial cell factories for efficient PHB biosynthesis. In addition, artificial intelligence and machine learning are expected to support strain design and bioprocess optimization, contributing to more efficient and sustainable PHB production. Genetic modification of PHB-producing microorganisms may also enable the biosynthesis of tailored copolymers with improved flexibility, thermal stability, and biodegradation performance [87,156].

### 11.3. Advanced PHB Composites

Future material development will likely focus on multifunctional PHB composites reinforced with natural fibers, nanofillers, compatibilizers, bio-based plasticizers, and polymer blends. Improved filler dispersion and interfacial compatibility may significantly enhance toughness, thermal resistance, barrier performance, and mechanical strength [12,17,75,118]. In particular, bio-based plasticizers such as glycerol trilevulinate (GT) appear promising for PHB matrices because they can improve melt processability and toughness while supporting the development of more sustainable PHB-based materials [124]. Future research should also focus on designing multifunctional PHB composites with improved mechanical and thermal performance while maintaining material sustainability. Such developments could broaden the use of PHB in packaging, biomedical, and engineering applications [157].

### 11.4. PHB in Additive Manufacturing

Additive manufacturing technologies such as FDM and FFF present strong opportunities for PHB-based materials. Future research should prioritize filament consistency, printability, dimensional stability, interlayer adhesion, and thermal control to minimize warping and degradation during processing [13,111,124,152]. Advancing multifunctional PHB composites for additive manufacturing with improved printability, mechanical performance, thermal stability, and biological functionality may also expand the application of PHB in biomedical engineering and other advanced manufacturing fields [158].

### 11.5. Circular Economy and Sustainability

Because PHB is bio-based and biodegradable, it has strong potential to contribute to circular economy systems. Future sustainable production pathways should integrate renewable energy, waste valorization, biodegradable composite design, and environmentally responsible processing technologies to further reduce life-cycle impacts [1]. Future PHB research should evaluate environmental impacts and economic feasibility alongside process development rather than treating them as separate issues. Combining life cycle assessment (LCA) with techno-economic analysis (TEA) can reveal where the main environmental and cost-related trade-offs occur, identify opportunities for process improvement, and provide a stronger basis for scaling up PHB production and commercialization [159].

### 11.6. Commercialization Potential

Although PHB-based materials have already entered commercial markets, broader industrial adoption remains limited by production cost, processing complexity, infrastructure constraints (e.g., using synthetic polymer equipment), and a general lack of working experience. However, increasing environmental regulations, technological improvements, and growing demand for sustainable materials are expected to accelerate PHB commercialization in packaging, biomedical, agricultural, and additive manufacturing sectors [19]. Although PHB-based products are already commercially available, their wider industrial use remains limited by high production costs, processing difficulties, inadequate infrastructure, and limited experience with large-scale manufacturing. Further commercialization will depend not only on improvements in production and processing efficiency but also on tailoring PHB properties to meet the requirements of specific applications. Data-driven methods could help identify the material characteristics that are most important for market performance and guide the development of PHB-based products for packaging, biomedical, agricultural, and engineering applications [19,160].

Overall, the future of PHB will depend on progress in biotechnology, materials development, manufacturing, sustainability assessment, and industrial scale-up. These areas should not be considered separately, since changes in production can directly affect material performance, processing, cost, and end-of-life behavior. A more connected approach that combines renewable feedstocks, improved microbial production, advanced PHB formulations, reliable manufacturing methods, and environmental and economic assessment could help make PHB more practical and competitive. Cooperation among researchers, industry, policymakers, and end users will also be important for addressing technical, regulatory, and market challenges and supporting the wider use of PHB in packaging, biomedical, agricultural, and engineering applications.

## 12. Conclusions

PHB is recognized as one of the most promising biodegradable biopolymers because of its renewable origins, biodegradability, biocompatibility, and material properties which are comparable to several conventional commercially available synthetic thermoplastics. This review comprehensively examined the production, biosynthesis, physicochemical properties, modification strategies, additive manufacturing potential, biodegradation behavior, and industrial applications of PHB-based materials.

Despite its strong environmental and functional advantages, the wider industrial use of PHB remains limited by brittleness, high crystallinity, narrow thermal processing range, thermal instability, and relatively high production costs. To overcome these limitations, modification strategies such as copolymerization, polymer blending, filler reinforcement, nanocomposite development, and plasticization have been widely investigated. These approaches have significantly improved the flexibility, toughness, thermal stability, melt processability, and printability of PHB-based systems. In particular, PHB composites and hybrid materials have shown promising potential for sustainable additive manufacturing through FDM technologies.

Biodegradation studies have shown that PHB can degrade under soil, marine, freshwater, composting, aerobic, and anaerobic conditions. Although PHB degradation may generate secondary microplastic particles, these particles can undergo further microbial degradation under suitable conditions. However, their degradation rate and environmental persistence depend on factors such as temperature, moisture, oxygen, and nutrient availability, microbial community composition, material formulation, and degradation time. In addition, life cycle assessment studies suggest that PHB may exhibit lower environmental impact than several conventional and bio-based plastics, further supporting its sustainability potential.

Although challenges related to cost, scalability, processing stability, and infrastructure still remain, continued advancements in biotechnology, polymer engineering, additive manufacturing, and sustainable production systems may significantly expand the industrial viability of PHB. Overall, continued advances in production, processing, material design, and end-of-life management may support the widespread use of PHB in packaging, biomedical, agricultural, environmental, and advanced 3D-printing applications.

## Figures and Tables

**Table 1:** Molecular characteristics of PHB synthesized by selected bacterial strains.

Bacterial Strain	MW (kDa)	MN (kDa)	PDI	References
*Bacillus megaterium*	200–800	-	1.2	[5]
*Cupriavidus necator* H16 (Lgg-16)	1411	1397	1.01	[90]
*Azotobacter vinelandii* N-15	1200	466	2.6	[91]

MW—Molecular weight; MN—Molecular number; PDI—Polydispersity index = MW/MN.

**Table 2:** Summary of thermal, structural, mechanical, and physical properties of PHB, PP, and selected biodegradable polymers [50].

Category	Thermal	Thermal	Structural	Mechanical	Physical	Mechanical
Property	*T*_m_ (°C)	*T*_g_ (°C)	Cr (%)	TS (*σ*) (MPa)	D (*ρ*) (g/cm^3^)	EB (*ε*) (%)
PHB	171–182	2–15	60	43	1.23–1.25	5
PHBV	137–179	−6–10	56	20	1.20	50
PP	171–186	−10	50–70	38	0.905	400
PLA	150–162	45–60	37	21–60	1.21–1.25	2.5–6
PGA	220–233	35–45	45–55	60–99.7	1.50–1.71	1.5–20
PCL	58–65	−65–60	54	21–42	1.11–1.15	300–1000

PHBV = poly-β-hydoxybutyrate-co-3-hydroxyvalterate; *T*_m_ = melting temperature; *T*_g_ = glass transition temperature; Cr = crystallinity; TS = tensile strength; D = density; EB = elongation at break.

**Table 3:** FDM/FFF processing parameters and filament fabrication conditions for PHB-based composite materials.

Material Blend	NT (°C)	BT (°C)	PND (mm)	FDT (mm)	LH (mm)	L/D Ratio	SS (rpm)	PS (mm/s)	Infill (%)
PHB/PHBV/PLA/PBAT [17]	190	30	0.4	1.6–1.8	0.2	25:1	30–50	40	100
PHB/PBAT/NFC [13]	210	60	-	2.85–3.00	-	-	45–50	1	-
PBS/PHB and PBS/PLA with CDI [105]	180	20	0.4	1.75 ± 0.05	0.2	-	30	40	-
PHB/PLA/corncob [116]	200	-	-	2.85	-	-	-	-	-
PHB/Starch filaments [119]	170	25 (unheated)	-	1.74 ± 0.10	-	-	2–35	15	20
PHB and microcrystalline cellulose powder [120]	185	Ambient (unheated)	-	2.85	0.1	42:1	2.5	-	100
PHB/GT [124]	180–200	60	0.4	1.55 ± 0.10	0.2	-	-	0.42	30
PHB/PLA/HA with plasticizer [125]	210	40	0.4	1.70–1.85	0.2	25:1	40–50	25	100
PLA/PHB/HDPE/PP [141]	-	-	-	1.75	-	-	-	-	-

NT—Nozzle temperature; BT—Bed temperature; PND—Print Nozzle Diameter; FDT—Filament Diameter Target; LH—Layer Height; SS—Screw speed; PS—Print speed; (-) not reported.

**Table 4:** List of global industrial companies manufacturing PHB on a commercial scale.

Monopolymer and Copolymer	Trade Name	Industry Name	Location
PHB	Biogreen^®^	Mitsubishi Gas Chemical Company Inc.	Japan
PHB	Biocycle^®^	PHB Industrial Company	Brazil
PHBV, PHBV + Ecoflex blend	Enmat^®^	TianAn Biologic Materials Co., Ltd.	China
P(3HB-co-4HB)	Green Bio	Tianjin GreenBio Materials Co., Ltd.	China
PHBH	Nodax™	Procter & Gamble (P&G)	USA
PHB	Mirel™	Telles (US)	USA
PHA	VersaMer™	PolyFerm	Canada
PHBV and PHB (Biopol)	Biomer^®^	Biomer Inc.	Germany
PHBH	Kaneka PHBH	Kaneka Corporation	Japan
BiomeHT 90	BiomeHT	Biome Technologies PLC	UK

## Data Availability

No new data were created or analyzed in this study. Data sharing is not applicable to this article.
